# The role of B cells in COVID-19 infection and vaccination

**DOI:** 10.3389/fimmu.2022.988536

**Published:** 2022-08-30

**Authors:** Shiru Chen, Fei Guan, Fabio Candotti, Kamel Benlagha, Niels Olsen Saraiva Camara, Andres A. Herrada, Louisa K. James, Jiahui Lei, Heather Miller, Masato Kubo, Qin Ning, Chaohong Liu

**Affiliations:** ^1^ Department of Pathogen Biology, School of Basic Medicine, Tongji Medical College, Huazhong University of Science Technology, Wuhan, China; ^2^ Department of Internal Medicine, The Division of Gastroenterology and Hepatology, Tongji Medical College, Huazhong University of Science and Technology, Wuhan, China; ^3^ Division of Immunology and Allergy, Lausanne University Hospital and University of Lausanne, Lausanne, Switzerland; ^4^ Institut de Recherche Saint-Louis, Université de Paris, Paris, France; ^5^ Laboratory of Human Immunology, Department of Immunology, Institute of Biomedical Sciences, University of São Paulo (USP), São Paulo, SP, Brazil; ^6^ Lymphatic and Inflammation Research Laboratory, Facultad de Ciencias de la Salud, Instituto de Ciencias Biomedicas, Universidad Autonoma de Chile, Talca, Chile; ^7^ Centre for Immunobiology, Bizard Institute, Queen Mary University of London, London, United Kingdom; ^8^ Cytek Biosciences, R&D Clinical Reagents, Fremont, CA, United States; ^9^ Laboratory for Cytokine Regulation, Center for Integrative Medical Science (IMS), Rikagaku Kenkyusho, Institute of Physical and Chemical Research (RIKEN) Yokohama Institute, Yokohama, Kanagawa, Japan; ^10^ Department of Infectious Disease, Tongji Hospital, Tongji Medical College, Huazhong University of Science and Technology, Wuhan, China

**Keywords:** B cells, antibody, COVID-19, SARS-CoV-2, memory B cells, vaccination

## Abstract

B cells secrete antibodies and mediate the humoral immune response, making them extremely important in protective immunity against SARS-CoV-2, which caused the coronavirus disease 2019 (COVID-19) pandemic. In this review, we summarize the positive function and pathological response of B cells in SARS-CoV-2 infection and re-infection. Then, we structure the immunity responses that B cells mediated in peripheral tissues. Furthermore, we discuss the role of B cells during vaccination including the effectiveness of antibodies and memory B cells, viral evolution mechanisms, and future vaccine development. This review might help medical workers and researchers to have a better understanding of the interaction between B cells and SARS-CoV-2 and broaden their vision for future investigations.

## 1 Introduction

The new coronavirus, severe acute respiratory syndrome coronavirus 2 (SARS-CoV-2), have caused over 550 million infections and more than 6 million deaths till July 18, 2022. The Chinese Center for Disease Control and Prevention identified the virus as a novel coronavirus on January 7, 2020 and reported the results of pathogen identification to the World Health Organization (WHO) on January 9, 2020. Then, more importantly, the genome sequence of this novel virus was registered in the global influenza sharing database on January 12, 2020 (1). WHO declared this outbreak a public health emergency of international concern as an alarm to the countries around the world on January 23, 2020 and named the infectious coronavirus disease COVID-19 (2). Bats are the likely reservoir hosts for SARS-CoV-2, while other species are intermediate hosts that harbor the virus to allow the replication and thus the mutations required to become highly transmissible and pathogenic to humans (3, 4). At present, targeted and effective vaccines have been under production and inoculation in many countries, and facilitated the prevention and control of the pandemic.

SARS-CoV-2 belongs to the Coronaviridae family, in which 229E, HKU1, NL63, and OC43 have caused small endemic infections, as well as the unanticipated worldwide outbreaks of SAR-CoV and MERS-CoV (5, 6). The human coronaviruses that are responsible for numerous chronic or acute respiratory diseases ranging from the self-curable common cold to severe pneumonia, contain an envelope and have single-stranded positive RNA genomes (7, 8). The genome of SARS-CoV-2 encodes four structural proteins including membrane (M), envelope (E), nucleocapsid (N), and spike (S). The S protein allows the virus to infect cells and mutations on this protein help the virus escape from existing neutralizing antibodies. Additionally, nonstructural proteins (NSPs) are also necessary and important for virus infection (9, 10). The spike protein contains the receptor-binding domain (RBD) and along with the nucleocapsid, acting as effective antigens that elicit B cell-mediated antibody response (11). Studies on severe COVID-19 cases have reported a greater antibody response against the S and N protein and larger memory B cell response towards S protein, suggesting that the more severe COVID-19 infection might provide superior protection from re-infection with SARS-CoV-2 (12).

In this review, we discuss the function of B cells in SARS-CoV-2 viral infection and reinfection. From the antibodies B cells secrete to other subsets like memory B cells and regulatory B cells, from the role of antivirus response to pathological impairment, and from natural infection to vaccination.

## 2 Overview of the immune system and the general role of B cells

In humans, there are two types of immune responses against infections that include the innate immune system and the adaptive immune system. B cells play a significant and irreplaceable role in the adaptive immune response, which contributes to the control, destruction, and clearance of invaders like viruses and bacteria. The role of B cells in virus infection is dynamic and far-ranging, involving cytokine production, antigen presentation, and antibody secretion.

B cells develop through the pro-B cells, pre-B cells, and immature B cells into mature B cells in the fetal liver before birth and afterwards in the bone marrow. Some mature B cell subpopulations, such as B-1, B-2, and regulatory B cells, are necessary components in antiviral B cell responses. B-1 cells derived from fetal liver could be divided into B-1a and B-1b subpopulations (13). B-2 cells originating from bone marrow contain follicular B (FO B) cells and marginal zone B (MZ B) cells. In general, MZ B cells and B-1 cells participate in T-independent responses to produce short-term immunity, while FO B cells are involved in T-dependent responses to produce long-standing protection against reinfection with the same pathogen (14). However, studies revealed that MZ B cells can not only elicit early-generated antibody responses but also play a role in germinal center (GC) formation with high similarity compared to FO-derived GCs except for a delay in T-dependent responses (15). Regulatory B cells function as immunosuppressive cells to support immunological tolerance by producing pro-inflammatory factors like interleukin-10 (IL-10) to inhibit the multiplication of T cells and other pro-inflammatory cells (16).

## 3 B cells response in COVID-19 infection

### 3.1 Humoral immunity

Humoral immune responses are the most effective and long-lasting immunological mechanisms for the clearance and prevention of reinfection and are critical for SARS-CoV-2 infected individuals. Limited humoral responses lead to ineffective clearance of SARS-CoV-2 in patients with immune deficiency, thus recidivation occurs (17).

**Figure 1:**
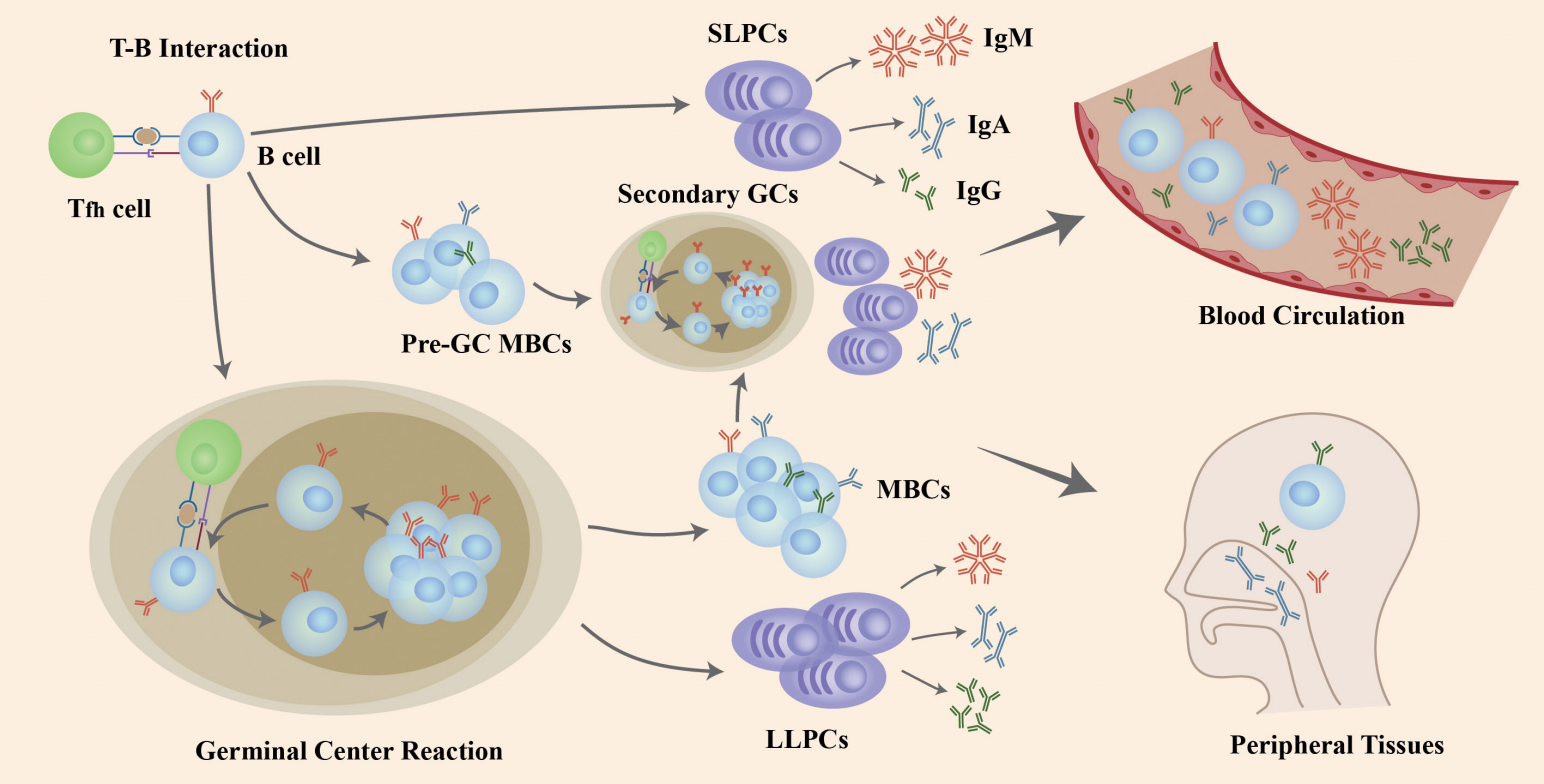
The role of B cells in COVID-19 infection and re-infection. Naïve B cells are activated *via* the help of folliculat T (Tfh) cells after the invasion of SARS-CoV-2 virus. Following T-B interaction, B cells go through rapid proliferation and differentiation, producing pre-germinal center memory B cells (pre-GC MBCs) and short-lived plasma cells (SLPCs) producing early antibodies with low affinity. Other B cells enter the germinal center (GC) to increase antibody affinity by clonal expansion and somatic hypermutation (SHM), and change the structure through class-switching recombination (CSR), resulting in long-lived plasma cells (LLPCs) and memory B cells (MBCs). If challenged with a second invasion or vaccination, both pre-GC MBCs and MBCs generated after GC reactions differentiate into plasma cells or generate a second GC in a short period of time to produce high-affinity antibodies. Those B cells and antibodies secreted enter the blood circulation and peripheral tissues like mucosa to defend against viral infection and re-infection. Figure adapted from (18).

The interaction of naïve B cells with the antigen and CD4+ T cells in GCs induces B cells to go through the process of proliferation, class-switch recombination (CSR) to affinity maturation and, eventually, differentiation into LLPCs and MBCs that produce antibodies (Abs) with high-affinity (**Figure 1**). After the T-B interaction, some B cells generate extrafollicular reactions in which its, rather than forming GCs, rapidly proliferate and differentiate into SLPCs that secrete low-affinity Abs and pre-GC MBCs that take part in the differentiation into plasmablasts or the initiation of secondary GC responses together with MBCs generated in GCs. T follicular helper (Tfh) cells activate and regulate the activity of B cells in the GC response (19) (**Figure 1**).

### 3.2 Primary immune response in SARS-COV-2 infection

#### 3.2.1 Functional GC response and the LLPCs generation

After GC response, the LLPCs migrate and locate in bone marrow and could produce neutralizing Abs continuously to protect individuals from re-infection. One study has found that the SARS-CoV-2-specific LLPCs could exist 7 to 11months in bone marrow and provide effective protection in mild COVID-19 infections (20). Moreover, investigations show that mRNA vaccines could induce persistent germinal center reactions, resulting in the unremitting production of LLPCs and the neutralizing Abs (21, 22). These might be the mechanism of the long-lived immune memory in mild-infected cases. In severely infected individuals, the elevated antibody titers and MBCs response correlate with the robust extrafollicular response rather than GC response (23–25). This might be attribute to the failure in functional GC formation due to the significant decrease in the Tfh cell quantity, resulting in the defective LLPCs and MBCs generation from GC response and impaired long-term protection (26).

#### 3.2.2 Antibody generation and response

After activation, B cells differentiate into antibody-producing cells and secrete Abs as a powerful weapon against primary or secondary infection (27). In SARS-CoV-2 infection, data show that neutralizing antibodies are generated from naïve B cells and not from pre-existing memory B cells (28–31). The affinity of Abs improves by SHM and clonal evolution in GCs to increase the binding efficiency to pathogens (32). Abs are generated into different isotypes that have diversity in the structure of the heavy chain constant region (CH), including IgA, IgG, IgM, IgD and IgE. This transformation of isotype is achieved through CSR (33) (**Figure 1**). Activation-induced cytidine deaminase (AID) is critical in the genomic recombination for SHM and CSR (34) and AID-deficiency is associated with hyper-IgM syndrome in patients (35).

Antibodies of COVID-19 patients bind S proteins and inhibit entry of viruses into host cells by sterically blocking the interaction of the receptor-binding domain (RBD) with ACE2 (36–38). While RBD remains the dominating target for neutralizing antibodies, studies reveal that other epitopes on the S proteins, like the N-terminal domain (NTD), also interact with virus-neutralizing antibodies (39, 40). However, one study indicates that the N protein has little reactivity to neutralizing antibodies and that antibodies binding non-S proteins provide little protection against COVID-19 infection (31).

Generally, in virus infections, the generation of serum IgM is dominant over other antigen-specific antibodies including IgA and IgG (41). However, the serum antibody production of IgM, IgA, and IgG against SARS-CoV-2 S and N proteins occur almost simultaneously in COVID-19 patients (42, 43). Studies further illustrate that IgA contributed more in neutralizing viruses than IgG and dominated the humoral response specific to SARS-CoV-2 in the early stage of COVID-19 infection in mucosa (44).

The heterogeneity observed in patients’ disease severity, humoral immunity, B cell response and antibody titers is associated with age, gender and race (45–47). Meanwhile, the severity of the disease is also highly associated with the load of antigens and the corresponding neutralizing antibody (nAb) titers, with the sickest patients having high load of virus, inducing high nAb titers and the milder or asymptomatic individuals having low or no nAb titers because of low antigen load (48, 49). These results are also observed in SARS and MERS infection (50). This suggests that a high nAb titer does not necessarily mean better humoral protection against the virus. Moreover, the quick decline of SARS-CoV-2 specific nAb titers (within 2 months) among severe, mild and asymptomatic patients further indicates that the protection from the humoral response is not only time-limited, but it is also insufficient (51, 52). Furthermore, the inconsistency between antibody response and disease outcome shows that some individuals resolve the virus infection before antibody production, indicating that the innate immunity and early T cell response may be the factors in determining the duration and severity of primary SAR-CoV-2 infection (53).

Antibodies prevent pathogens from entering host cells and they also agglutinate free viruses and kill virus-infected cells (49). Although it remains to be shown in human COVID-19 cases, these significant mechanisms have been studied in mice (52, 54). These models have shown that antibodies with high Fc effector activity provide the most protection against SARS-CoV-2 infection (52), and that vaccination using RBD-Fc based activity was effective in fighting COVID-19 infection (54). Additionally, human patients that have died of COVID-19 produced antibodies with decreased Fc effector activity, which also resulted in deficient humoral induced immune responses (55). Altogether, these results provide a new approach in developing efficient and broad-spectrum vaccines involved in protection against SAR-CoV-2 re-infection. However, even minor changes to the Fc structure of antibodies, like IgG that contains fucosylated Fc glycans, may negatively influence Fc-related interaction. This could result in increased secretion of inflammatory cytokines and disease severity which resembles what has been observed in severely infected patients (56). Although the treatment of COVID-19 with antibody Fc appears to be a promising therapy, further studies are needed.

Apart from the function of antibody secretion, B cells also play an important role in inflammatory cytokines. The expression of several inflammatory cytokines such as TNF-α, Interleukin-2, Interleukin-6, monocyte chemoattractant proteins (MCPs), and IFN-γ-inducible protein 10 (IP-10) as well as relevant chemokines such as CXCL2 and CCL2 are elevated in COVID-19 patients, accounting for the cytokine storm observed in these patients (57–59). However, the mechanism of the cytokine storm formation and amplification remains to be unknown, and more investigations are needed to explore it in order to give proper suggestion on clinical treatment and therapy.

### 3.3 Secondary response in SARS-CoV-2 infection

#### 3.3.1 Memory B cell subsets

Memory B cells (MBCs) take an important part in deciding the occurrence and severity of COVID-19 re-infection, thus finding ways to elicit widely reactive MBCs is key in vaccine development. MBCs develop through either GC-dependent or GC-independent pathways (60) and are classified into different subsets with distinct functions and markers (61, 62). One atypical MBC is characterized by CD11c^+^ T-bet^+^, develops independently from GCs, and plays a protective role in COVID-19 infection (25, 63, 64). In general, MBCs that do not undergo GC response lack class switching and affinity maturation and are incapable of producing effective nAbs against viral variants (60, 65, 66). Additionally, CD80-PD-L2 double negative population of MBCs do not go through GC reaction and differentiate into antibody-secreting cells in a short period of time. However, they are able to re-enter GCs for activation and development into antibody secreting cells that produce nAbs with high-affinity (66, 67). Therefore, methods to promote the re-entrance of MBCs into GCs to generate protective nAbs are important for creating potential therapies against COVID-19.

#### 3.3.2 B cell memory effector functions in COVID-19 infection

During COVID-19 re-infection, MBCs circulating throughout the whole body are activated and quickly differentiate into Ab-secreting cells. However, if these circulating Abs are deficient or activated by a variant of the primary pathogen, the MBCs are induced to generate new GCs for producing higher affinity Abs (68). The antigen-specific Abs are lost in the large majority of SARS-CoV-infected patients. This may be similar to what is seen in SARs patients, where specific MBC responses are undetectable in 100% of the recovered individuals 6 years after infection (69). Overall, the protection offered by immune memory against reinfection of coronaviruses only persists for a short period of time (70).

There are limited human studies on the memory immune response in COVID-19 re-infection. The common method to evaluate B cell memory is monitoring the amount and duration of circulating antibodies in serum. One study indicates that the antigen-specific IgG titers keep steady for 3-4 months after infection, while IgA and IgG are maintained for an even shorter time (71, 72). In contrast, many other studies have shown durability in immune memory. In one cohort study, 25 patients grouped as mild, moderate, or severe were assessed for RBD or N-specific MBCs and it was found that all groups had increased MBCs from the beginning to 150 days after infection (73). Additionally, the RBD- or N-specific MBCs mainly contain IgM and IgG with various immunophenotypes, and the quantity of RBD-specific IgG MBCs also correlates with the number of Tfh cells. In the early stage of re-infection, IgM secreting MBCs represent the majority of MBCs, but they decrease after 20 days as IgG secreting MBCs steadily increase and become the dominant MBCs that are detectable between 120-240 days post-symptom onset (PSO). As for long term studies, a cohort evaluation of 188 cases found that circulating MBCs specific for RBD, N and S proteins could exist for more than 6 months and up to 8 months PSO. Additionally, a large proportion of RBD memory B cells that increased over time from 1 month to 6 months PSO secreted IgG (24). Another longitudinal study investigated the amount of MBCs in mild cases and found that IgG^+^ MBCs persisted and increased through 3 months after COVID-19 infection (74). RBD-specific MBCs that have undergone SHM in GCs and produce high affinity nAbs have frequencies that remain unchanged or even rise 6 months after infection (75, 76). Moreover, a recent study showed an encouraging discovery that functional IgG MBCs persist after 5-8 months in infected patients, while the level of serum SARS-CoV-2 specific IgG is eventually lost (77). This might suggest that the level of MBCs is more sensitive for detection of previous infection and prediction of long-term protection. Overall, these results show that the B cell-mediated humoral response, which produces short-term or long-term MBCs and antibodies, provides indispensable protection during reinfection. Clinical findings also support the research above, with nearly 1200 individuals showing no symptoms during COVID-19 reinfection (78). However, due to the limited re-infected populations and insufficient information, more researches are required to further investigate the time span and protective magnitude of B cell responses in reinfections.

### 3.4 B cell response in mucosal immunity

Respiratory viruses like SARS-CoV and SARS-CoV-2 enter organisms mainly *via* the mucosal surfaces of the respiratory tract. Antibody and B cell responses are critical in controlling virus adherence and invasion effectively in the peripheral tissues. Collecting blood samples to assess immune responses is the most common method because it is the most direct and convenient way, but blood samples do not represent the immune cells and response in peripheral tissues, like mucosa (79). Thus, it is important to understand the relationship between B cells and antibody response not only in blood, but in tissues too.

#### 3.4.1 Antibody response

Currently, less is known about mucosal immunity against SARS-CoV-2 compared to systemic immunity. Both IgA and IgG are produced in mucosal tissues. IgA dimers specific to SARS-CoV-2 are nearly 15 times more potent than IgA monomers, which play a major role in neutralizing viruses and preventing initial viral spread and amplification (80). One study detected the level of antibody responses in both serum and saliva and described that the IgG response was similar and lasted for several months while the titers of IgA and IgM declined rapidly (81). Further investigations revealed that there is a positive correlation of IgG and IgM responses between blood and mucosa, while the relation with IgA is less comparable (81, 82). In nasal samples collected from COVID-19 patients, severely infected individuals had higher IgG responses while those with mild or moderate symptoms showed higher IgA responses (83). These findings on mucosal immunity may not be universally applied in all cases, but they are evidences that B cells and antibody responses are playing a critical role in immune mechanisms against SARS-CoV-2 and may influence the outcome of infection.

#### 3.4.2 Memory B cell response

Although existing antibodies in mucosal tissues can prevent humans from pathogen infections like COVID-19, MBCs play an important role during reinfection by responding locally to increase production of antigen-specific antibodies in a short period of time. MBCs are present in multiple human tissues, such as lung, spleen, lymph nodes and gut (84–87). MBCs in the lung enhance immune responses against viruses and provide cross-reactive antibodies through clonal selection in germinal centers (85–88). In the gut, MBCs enable continued clonal selection in mucosal to generate B cells which secrete antigen-specific antibodies with high affinity (75). MBCs in peripheral tissues, like lung, require tertiary lymphoid structures induced by inflammation to undergo reactivation and produce antibodies to clear viruses rapidly (89, 90).

The development of a vaccine that induces mucosal B cell responses to generate SARS-CoV-2 specific antibodies and relevant memory B cells are of great significance to prevent and protect against viruses. One investigation revealed that even individuals fully vaccinated with mRNA vaccines could still be infected by SARS-CoV-2 variants (91). The level of IgA and IgG in saliva is much lower than in serum, accounting for the ineffective protection in mucosal compartment induced by intramuscular vaccines (92). However, animal studies have shown that a single dose of the adenovirus-vectored SARS-CoV-2 vaccine had better protective effects in upper and lower respiratory tracts in mice and hamsters compared to other vaccines (93, 94). Further investigation confirmed that this adenovirus-vectored SARS-CoV-2 vaccine could provide general mucosal immunity against SARS-CoV-2 and the variants (95). A phase I/II clinical trial of a novel vaccine, NASVAC, was designed to measure its effectiveness in inducing mucosal immune response in COVID-19 patients *via* intranasal approach, declaring the safety and tolerance of this vaccine to infected patients (96). Other studies also found that an intranasal approach of vaccination and antibody therapies could be effective to protect individuals against COVID-19 infection (97, 98). Overall, the existence of memory B cells and secreted Abs in mucosal tissues provide important goals in vaccine development since the response of mucosal immunity measures the protective level in uninfected individuals and the disease outcome in infected patients (99).

## 4 B cell response in vaccination

The rapid spread and frightful mortality of SARS-CoV-2 sounded a global alarm and the research and development for an effective vaccine to control the epidemic became an emergency.

Several different types of vaccines have been used worldwide. At first, two inactivated vaccines including BBIBP-CorV and CoronaVac and one RBD-based protein subunit vaccine, ZF2001, were authorized for wide use (100–103). Three clinical trials in phases I and II, which included the candidate vaccine, CoronaVac, revealed effective production of neutralizing antibodies with low rates of adverse reactions in three healthy age groups of 3 to17-years old, 18 to 59-years old and more than 60 years old (100–102). Other two studies focusing on the safety and effectiveness of BBIBP-CorV revealed similar outcomes, with most individuals having a humoral response in 28 days post-vaccination with no severe adverse events (103, 104). However, one study that was randomized, double-blinded and placebo-controlled showed that ZF2001, the RBD-based protein subunit vaccine, caused more and worse side-effects compared to the other two vaccines, but still was beneficial in protection against COVID-19 (105).

Later, mRNA-based BNT162b22 (Pfizer-BioNTech), mRNA-1273 (Moderna) and viral vector-based Johnson & Johnson Janssen vaccine were authorized by the Food and Drug Administration (FDA) for emergency use. In the individuals who were not infected before, mRNA-based vaccines induced B cell responses and antibody generation effectively, enabling acquired immunity against SARS-CoV-2 infection (53, 106, 107). Half of the vaccinated individuals developed detectable nAbs with one dose and the majority generated adequate antibodies and enhanced B cell memory after a second dose (106, 107). In addition, the first dose of both Pfizer-BioNTech and Moderna vaccines induced anti-S IgG titers that declined after 6 weeks, thus further confirming the necessity of a second dose (53). These two mRNA-based vaccines also promote a protective B cell immune response in individuals that recovered from COVID-19 infection (65, 107–109). With these individuals, after the first dose, vaccination successfully induced an enhanced memory B cell response with neutralizing antibodies specific to SARS-CoV-2 (108). Also, the number of existing memory B cells all increased after the vaccination and the degree of enhancement correlated with the number of memory B cells pre-existing in the SARS-CoV-2 infected individuals (65, 107). However, the second dose did not further increase the B cell memory antibody response, suggesting that only the first dose of the mRNA-based vaccines is helpful in reaching the peak of humoral immunity in recovered SARS-CoV-2 individuals (109). While the quantity of memory B cells in recovered individuals was higher than those in uninfected individuals at 3 months post-vaccination, the number of memory B cells at 6 months and the decline rate afterward remained similar (110). Furthermore, several studies showed that mRNA-based vaccines induce cross-reactive nAbs against SARS-CoV-2 variants in not only naïve but also recovered individuals (65, 111).

One article revealed that most vaccines are safe even in cancer patients, but a customized vaccination approach is preferred (112). While the vaccines above have been proven safe in clinical investigations, they are insufficient to provide enough protection for this universal outbreak, therefore, creating new vaccines with diverse protective mechanisms is necessary. A study of one influenza virus vaccine, which can alternatively induce stalk-reactive memory B cells in comparison with conventional influenza virus vaccines, has completed the phase I trial and has proven safe and efficient in generating a more prolonged and broader B cell and antibody response (62). Targeting the conserved and immunodominant antigen of hemagglutinin, this vaccine has a novel approach to vaccine production, but whether this finding can be applied to making SARS-CoV-2 vaccines is unclear because of the less protective cross-reaction between SARS-CoV-2 and seasonal coronaviruses (31).

## 5 SARS-CoV-2 variants

Coronaviruses, like SARS-CoV-2, have a decreased mutation rate compared to other smaller RNA viruses because of their 3′-5′ exoribonuclease, which has an efficient proofreading function in mismatch correction (113, 114). However, various mutations with enhanced pathogenicity, transmissibility, or escaping capability from neutralizing antibodies have been reported in new waves of epidemics around the world.

An early study following the viral genome evolution of the Wuhan-Hu-1 strain after one year found 75 non-synonymous nucleotide mutations in 3,823 samples compared to the initial viral copy (EPI_ISL_402125) from January 2021 (115). The viral variant D614G, with a mutation in the nucleotide fragment encoding the spike protein, was more infectious and rapidly replaced the former Wuhan-Hu-1 during the early stage of the COVID-19 epidemic (116). Ever since then, several changes in the amino acid of the S protein have been identified and variants with increased spread and escape from vaccination and natural immunity are emerging (117). Studies also showed that the sensitivity of neutralizing antibodies to the spike proteins has decreased against several virus variants (9, 118–121).

Consequently, vaccination for inducing effective antivirus immunity including MBCs and nAbs have become significant in protecting against infection or reinfection by SARS-CoV-2 variants. One dose of mRNA vaccine is sufficient to elicit enhanced antibody responses with higher antibody titer specific to Alpha (B.1.1.7) and Beta (B.1.351) variants in previously infected patients (107). But there needs to be two doses to induce enough nAbs against the S protein of Alpha, Beta and Delta (B.1.617) variants (107, 108, 122). The mutation E484 was especially adept at escaping neutralization by antibodies in vaccinated individuals (123, 124). Interestingly, the titer of antibodies against Beta variants was not reduced in vaccinated individuals who were previously infected by SARS-CoV-2 (65, 107). Antibodies specific to both wild-type and viral variants from those vaccinated who were previously infected showed an increase in neutralizing titer in comparison to naïve individuals vaccinated (107, 125). No significant increase was found in the quantity of MBCs against variants post vaccination in previously infected individuals (65). These results suggest that vaccinated individuals with previous COVID-19 infections have more intensive humoral responses compared to those individuals who remained uninfected. This might also be due to clonal evolution increasing the breadth of B cell responses after infection (65, 126). Also, that could be an explanation of some breakthrough infection caused by delta variant with low frequencies of the memory B cells resulted from the impaired clonal evolution, neutralizing potency and neutralizing breadth (127). Memory B cells undergo clonal evolution persistently after at least one year of infection and secreting antibodies specific to Alpha, Beta and Delta variants were important in preventing reinfection and influencing disease outcomes in severely infected patients (65, 110). Consistent with the research above, there were 10 in 15 MBC clones at 12 months generating antibodies capable of neutralizing all tested variants while only 1 of 15 clones at 1.3 months (65), which further proves the critical role of MBCs in protective immunity mechanisms in infection and reinfection. The fifth SARS-CoV-2 variant, Omicron, was reported on Nov 25, 2021 and characterized by the high resistance against the antibodies induced by infection and vaccination (128, 129). In symptomatic infection caused by the omicron variant, two doses of BNT162b2 or ChAdOx1 nCoV-19 became mostly ineffective 6 months after vaccination (130, 131). Three doses of BNT162b2 might be more protective, however, antibody neutralization evasion still exist in omicron infected disease (129). The neutralizing Abs induced by mRNA vaccination in COVID-19 patients with primary antibody deficiency (PAD) were found to have limited protective function against Omicron variant (132). Studies also indicated that this newly generated variant, especially its spike protein, could evade most therapeutic antibodies (129). Therefore, improvement in vaccination appliance and clinical treatment is of great emergency.

## 6 Pathological B cell response

The humoral response is important in defending against viral infections such as COVID-19, but the damage to the host by its own B cells also needs to be considered to have a better understanding of the immune system and the mechanism in COVID-19.

### 6.1 Double-negative B cells

Double-negative (DN) B cells are peripheral B cells which have gone through maturation but lack of IgD and CD27 expression (133). Former studies have shown the association between DN B cells and rheumatoid arthritis, systemic lupus erythematosus (SLE), HIV, and other immunopathological diseases, in which DN B cells expand, release cytokines and produce autoimmune antibodies that enhance disease progression (134–136). Recently, one study showed that DN B cell subsets are associated with the severity of COVID-19 infection (137), but it is unclear the specific role of each subset and the mechanism of DN B cells in the SAR-CoV-2 outcome.

### 6.2 Early antibodies with antibody-dependent enhancement of disease (ADE)

While antibodies secreted by B cells are great weapons to fight SARS-CoV-2 and other pathogens, they could be harmful in some cases. Previous studies have shown that SARS patients who respond to viral infection early and produce detectable antibodies within two weeks have higher mortality than patients that are later responders (138). This same outcome has been observed in COVID-19 infected patients too (139). An explanation for this is that a skewed macrophage response increases a pro-inflammatory M1 phenotype, which enhances inflammation and impairs tissues in patients who generate early antibodies, especially IgGs against spike protein (140). This is known as an antibody-dependent enhancement of disease (ADE), which also occurs in SARS-CoV and MERS-CoV infections (141, 142). ADE develops when early produced antibodies promote viral entry into macrophages and other innate and adaptive cells *via* binding to both the spike protein of viruses and the Fc receptor on cell surfaces to form a functional complex (142). The timing of an antibody response is significant in antibody-based therapies, in which the outcome for patients is influenced by the timing of treatment application. However, there are no methods to differentiate viral infections from ADE and the risk of ADE is unpredictable, therefore the possibility of ADE-antibodies occurring in vaccinated individuals is of concern (143). Interestingly, there is no evidence of ADE during infection of COVID-19 vaccinated individuals by escape variants.

### 6.3 Autoimmune antibodies

Autoimmune antibodies have been reported in SARS-CoV-2 infected individuals. However, there is no conclusion whether these antibodies are common, short-lived types from anti-viral responses or pathogenic autoantibodies that cause autoimmune diseases like rheumatic heart disease (144). A particular B cell-mediated autoimmunity that lowers serum levels of IFNs by producing neutralizing IgG against type I IFNs was found in at least 10% of patients with life-threatening COVID-19 pneumonia (145). Additionally, a group of severe COVID-19 patients also suffered from worse symptoms due to an inborn error of type I IFNs (146). These two factors of lower IFN levels in patients cause devastating disease because of insufficient natural and acquired immunity, which indicates that the preexistence of autoimmune antibodies increase the severity of COVID-19 infection. Consistent with the hypotheses, these autoantibodies neutralizing type I IFN were also founded in nearly 20% COVID-19 deaths (147, 148). Samples from more than 34,000 uninfected individuals were analyzed and showed that the level of these autoimmune antibodies increased with age, which account for the risk of severe COVID-19 infection cases associated with age (147). Furthermore, a previous investigation on a yellow fever live attenuated vaccine revealed that more than 10% of vaccinated cases had a high titer of autoantibodies against type I IFNs, which explained the adverse effects in more than half of the life-threatening vaccine-associated disease (149). The connection between autoantibodies and vaccine side effects is a cause of concern in SARS-CoV-2 vaccination, thus further research is necessary to uncover the linkage and mechanisms involved.

Numerous case reports showed that *de novo* autoimmune disease would develop following COVID-19 infection, such as rheumatoid arthritis, psoriatic arthritis, and predict a worse disease outcome and prognosis (150–153). The most common antibodies are screened in SARS-CoV-2 patients, in which antinuclear antibodies, antineutrophil cytoplasmic antibodies, and ASCA immunoglobulin A antibodies showed great prevalence (153). Another functional antibody, antiphospholipid autoantibodies (aPLs), which cause abnormal coagulation along with microvascular and macrovascular thrombosis, is found in severe COVID-19 infected patients as well as Antiphospholipid syndrome (154, 155). Antiphospholipid syndrome is an autoimmune thromboinflammatory disease associated with aPLs, which stimulate endothelial cells and platelets activation and neutrophil extracellular traps (NETs) release from neutrophils (156–158). More than half of hospitalized SARS-CoV-2 infected patients had the presence of aPLs in serum samples. Moreover, the higher levels of aPLs have a positive association with neutrophil activation, NETs release, platelet quantity, and COVID-19 disease severity (144). All of these antibodies might be generated by a subpopulation of extrafollicular B cells, known as DN B cells which we have discussed before (25). These studies suggest that COVID-19 infection could trigger the autoantibody production, resulting in the *de novo* autoimmune disease and life-threatening infection.

### 6.4 Immune evasion

Viruses have developed multiple ways to escape the immune system, including SARS-CoV-2, which is likely to escape early neutralizing antibodies *via* antibody-driven evolution and other mechanisms that have not been discovered yet. One mutation, D614G, enhanced furin-mediated spike cleavage to increase syncytium formation and viral titer, which caused the virus to become more infectious with higher viral loads compared to the former viruses (159–161). Other recent variants, for example, B.1.351, B.1.1.7 and P1, became resistant to neutralizing antibodies by changing infected host cell reactions to escape immune clearance, which allowed for re-infection of recovered individuals and new waves of COVID-19 (121, 162). However, specific mutations that may have the greatest antigenicity and pathogenicity remain unclear. Therefore, understanding viral evolution mechanisms and preferences is extremely significant in vaccine design.

Antigen presentation is the initiation process for adaptive immune response (163). The downregulation of major histocompatibility complex (MHC) I and II molecules and pathways in antigen presenting cells, such dendritic cells and B cells, suggested that the antigen presentation was inhibited by SARS-CoV-2, leading to the inhibition of T cell-mediated immune response (164, 165). T cells not only participate in T cell immune response against SARS-CoV-2 (166), but also various B cell-associated immune responses, such the T-B interaction and cytokine production (167). Therefore, the impaired T cell activity would result in the less protective humoral and cellular response against SARS-CoV-2 as well as viral evasion from adaptive immunity.

## 7 Discussion and outlook

Multiple studies have been focusing on questions about the humoral response and the function of antibody-generating B cell and memory B cell responses. Several key problems still need answers such as: 1) the duration of antibodies and memory B cells in serum and mucosa, 2) the difference between the titers of neutralizing antibody and population of memory B cells in vaccination and infection, and 3) the factors influencing the outcome of disease in infected patients. Whether and how vaccination could induce an effective B cell response in mucosa requires further investigation. Current research has revealed that vaccination provides effective protection for naïve and previously infected individuals. However, with the ongoing viral evolution, vaccines also need to be continually updated and new approaches to defend against viral invasion are also urgently required for vaccine development, such as inducing effective mucosal immunity of dimeric IgA and memory B cells specific to SARS-CoV-2. Additionally, cross-reactive antibodies induced by infection or vaccination, which protects against not only COVID-19 but also other coronaviruses infections are important areas of study.

## Author contributions

SC wrote the article. FG, FC, KB, NOSC, AH, LJ, JL, HM, and MK revised the draft. CL and QN organized and revised the draft. All authors contributed to the article and approved the submitted version.

## Funding

This work was supported by HUST Academic Frontier Youth Team (2018QYTD10).

## Conflict of interest

The authors declare that the research was conducted in the absence of any commercial or financial relationships that could be construed as a potential conflict of interest.

## Publisher’s note

All claims expressed in this article are solely those of the authors and do not necessarily represent those of their affiliated organizations, or those of the publisher, the editors and the reviewers. Any product that may be evaluated in this article, or claim that may be made by its manufacturer, is not guaranteed or endorsed by the publisher.
